# Preconditions Contributing to Interprofessional Collaboration in the Management of COPD in Primary Care: A Scoping Review

**DOI:** 10.5334/ijic.8991

**Published:** 2025-12-26

**Authors:** F. L. De Zwart, E. W. M. A. Bischoff, L. Van Den Bemt, M. Perry, B. Van Den Borst, M. De Man, M. Van Den Heuvel, M. A. Spruit, A. J. Van ‘T Hul

**Affiliations:** 1 Department of Pulmonary Diseases, Radboud University Medical Center, 6525 GA Nijmegen, The Netherlands; 2 Department of Primary and Community Care, Radboudumc Research Institute for Medical Innovation, P.O. Box 9101, 6500 HB Nijmegen, The Netherlands; 3 Department of General Practice, Erasmus MC, University Medical Centre Rotterdam, P.O. Box 2040, 3000 CA Rotterdam, The Netherlands; 4 Department of Primary and Community Care, Radboud Institute for Health Sciences, Radboud University Medical Center, P.O. Box 9101, 6500 HB Nijmegen, The Netherlands; 5 Department of Pulmonary Diseases, Bernhoven, 5406 PT Uden, The Netherlands; 6 Department of Respiratory Medicine, Maastricht University Medical Centre, NUTRIM Institute of Nutrition and Translational Research in Metabolism, Faculty of Health, Medicine and Life Sciences, Maastricht University, Maastricht, The Netherlands; 7 Department of Research and Development, Ciro+ B.V., Hornerheide 1, Horn, 6085 NM, The Netherlands

**Keywords:** Interprofessional, collaboration, primary care, COPD, Rainbow model

## Abstract

**Introduction:**

Interprofessional collaboration (IPC) has been proven effective for COPD patients, however an overview on how to develop and sustain IPC in primary care is lacking. The objective of this review was to identify preconditions for IPC in primary care COPD management. Secondary objectives were to study if the identified preconditions differed from those found in the general primary care setting and secondary and tertiary COPD setting.

**Methodology:**

Three separate searches were executed in four databases for publications reporting preconditions for IPC. The identified preconditions were categorised into the domains of the Rainbow Model for Integrated Care (RMIC).

**Results:**

The first search revealed 32 preconditions and covered all RMIC domains. In the second search, 12 additional preconditions were found, with 90% of preconditions overlapping with the first search. The third search revealed only one study and no extra preconditions were identified.

**Conclusion:**

Many preconditions need to be considered when developing IPC for COPD in primary care. However, these are not setting or disease specific. This makes it possible to develop IPC in primary care for multiple chronic conditions and using knowledge gained from other healthcare settings.

## Introduction

Chronic Obstructive Pulmonary Disease (COPD) is a highly prevalent chronic respiratory condition with a substantial disease burden [1,2,3]. To diagnose the presence of chronic airway obstruction, a relatively simple spirometry is sufficient [1,2,3,4]. However, to fully understand the impact and burden of an individual and to reveal relevant targets for therapy beyond pulmonary function, a comprehensive assessment is required [5,6,7]. Such a holistic assessment results in the identification of both pulmonary (such as bronchoconstriction and chronic sputum production) and extra-pulmonary (such as deconditioning, malnutrition and depression) treatable traits (TTs) and forms a basis for an individual comprehensive management plan [7,8,9,10,11].

Addressing these TTs adequately in treatment will often require multiple and different health care professionals (HCPs) because they often occur in plural and can interact with each other [7,8,12,13,14]. For example, an abnormally low fat-free mass is treated by a combination of strength training (supervised by a physiotherapist) and protein-enriched nutritional supplements (supervised by a dietician) [15].

Providing complementary care for a patient by two or more HCPs, based on a comprehensive treatment plan is called interprofessional collaboration (IPC) [16]. Another key feature of IPC is the patient being part of the team. In practice this means intensive involvement in goal(s) setting and decision making. Also coordination and alignment of care hallmarks IPC [16]. Continuing the previous example, this could mean scheduled consultations between dietician, physiotherapist, and patient.

IPC has the potential to make a difference for patients with COPD [12,17,18,19,20]. In the Netherlands, as in many other countries, IPC for COPD has successfully been applied in pulmonary rehabilitation programmes in secondary and tertiary care services [21,22]. However, it is estimated that less than 5% of all patients with COPD are referred to pulmonary rehabilitation [12,14,22,23,24,25]. This can be due to patient, HCP or systemic factors, like the obligation to quit smoking, lack of availability, or guideline non-adherence [23,26,27]. To broaden the reach of IPC for COPD, embedding these programmes in a primary care setting seems promising. Primary care is characterised by first-contact medical access, long-term person- (not disease) focused care, comprehensive care, and coordinated care [28]. Besides, most of the patients with COPD are diagnosed and treated in primary care [29]. A significant portion of these patients perceive a high disease burden, associated with the presence of multiple TTs, which makes them eligible for IPC [7,12,14,30]. However, until now, IPC for patients with COPD has hardly been implemented in primary care [29].

Addressing preconditions (i.e. barriers and facilitators) during an implementation process is essential for success [31]. Given the potential of IPC in the management of primary COPD care, the absence of an overview of preconditions is remarkable. Furthermore, it would be beneficial to ascertain whether IPC in primary care COPD management has the same conditions as other primary care IPC programmes, or whether, conversely, there is overlap with COPD IPC programmes in other healthcare settings, like secondary- and tertiary care. This information could help making an educated decision on the best strategy to facilitate the use of IPC in primary care COPD management.

Thus, the primary objective of this study was to identify which preconditions benefit IPC in the management of COPD in primary care. The secondary objective was to identify how these preconditions differed from those described in studies on IPC in (a) the primary care setting for the management of other chronic diseases or (b) COPD management in the secondary or tertiary care setting.

## Methods

### Study design

We conducted a scoping review based on the Prisma checklist (Appendix 1) and on Arksey and O’Malley’s methodological framework, including identifying relevant studies, selection of eligible studies, charting the data, and collating, summarizing and reporting the results [32,33]. This scoping review was used to summarize research findings and disseminate these to relevant HCPs and policy makers [32]. No critical appraisal of individual sources of evidence was carried out, which is common in scoping reviews [33].

### Identifying relevant studies

To address the primary aim and two secondary objectives, three separate literature searches were conducted with the support of a medical librarian. For the primary objective, search terms were: ‘preconditions’, ‘interprofessional’, ‘collaboration’, ‘primary care’ and ‘COPD’, including synonyms, related terms, MeSH terms, Boolean operators, and truncations, including all types of research (COPD primary care setting). For the secondary objective, two additional searches were formulated: search 2 (named: general primary care setting) includes all aforementioned terms except all terms related to COPD. If patients with COPD were part of the sample, these articles were not excluded. And search 3 (named COPD setting) includes all the terms, except those related to primary care. Only IPC-approaches in secondary or tertiary care or the combination of multiple settings were included in this search. These two additional searches were restricted to reviews as including all study designs would yield an unattainable number of articles. In the three searches, papers were excluded when an explicit description of a precondition or a description of IPC was lacking, the collaboration contained less than 3 professionals, the paper was an editorial, comment or protocol, or publications focussing on adolescents, young adults, or the paediatric population as this group differs much from the COPD population. Details of all three search strategies are given in Appendix 2.

All searches were conducted in Pubmed, MEDLINE, EMBASE and Web of Science (by LdZ) and covered the period from January 1^st^ 2013, until March 19^th^ 2024. Only papers in English and Dutch were included. The start date was based on the publication of the ‘2012 American Thoracic Society Statement’ on the importance of IPC in COPD care [25]. The records from the different databases were combined and de-duplicated using EndNote bibliographic software [34].

### Study selection

Titles and abstracts were screened independently by two researchers (LdZ and AvtH) using two electronic open-access tools: Rayyan and ASReview. We used Rayyan for under 250 hits per search, with ASReview above 250 hits. Rayyan facilitates independent screening in a secured online cloud. All titles and abstracts were assessed and marked as ‘include, exclude, or maybe’ in Rayyan, whereafter disagreements were discussed until consensus was reached [35].

ASReview is an AI-aided screening tool which facilitates screening of large numbers of citations yielded from sensitive search strategies [36,37]. The AI strategy and the described protocol minimalize the chance of missing a relevant article without screening all abstracts. ASreview sorts abstracts on relevance, at first based on 3–5 key papers on the subject of the study, and subsequently it constantly refines the order of the unread abstracts based on the coding by a researcher (‘relevant’, ‘irrelevant’ or ‘?’). The two researchers coded abstracts independently which led to a different screening order of unread abstracts [36,38]. We used the stopping criteria 1) screening at least 200 title/abstracts and 2) have a consecutive series of 50 titles/abstracts marked as ‘irrelevant’ [37].

Researcher LdZ repeated the screening procedure with different settings after the first stop criterion was reached. To rigour the abstract screening process, screening was continued until the stopping criterion was reached again. Settings and workflow can be found in Appendix 3. Title/abstracts assessed as relevant by both researchers were screened for full text. All title/abstracts considered relevant by only one researcher were reassessed together until consensus was reached.

Full texts were screened independently by researchers LdZ and AvtH. All articles were discussed and reasons for full-text exclusion were noted.

### Charting the data

Data were extracted from all included articles using a standardized extracting form, including: 1) title, author, year of publication and study aim, 2) type of study, 3) type of healthcare setting(s), 4) sample, and 5) preconditions of IPC. If an article contained multiple aims, only the relevant sections were extracted. Researchers LdZ and AvtH extracted the data of their half of the papers, followed by cross-checking by the other. All extracted preconditions were neutrally or positively (re)formulated as most could be interpreted in two-ways. For example, the barrier ‘lack of time’ was reformulated to ‘sufficient time’. LdZ and AvtH independently described the precondition texts to resemble the original as closely as possible and discussed these with a third researcher (EB) until consensus was reached.

### Collating, summarizing and reporting the results

**Figure 1:**
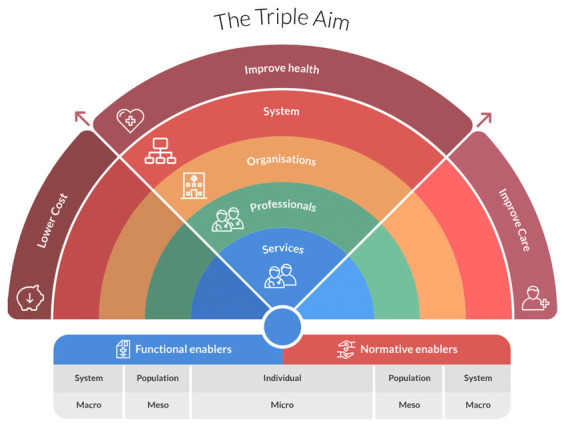
Rainbow Model of Integrated Care, by Pim Valentijn (2015), copyright 2017 (Essenburgh Group), Harderwijk, the Netherlands, used with permission [39,41,42].

In the analysis, we took a qualitative approach. In phase one, the extracted preconditions were inductively aggregated into groups by multiple researchers (LdZ, AvtH and EB). We invited a fourth researcher (MP) in phase two to prevent tunnel vision. The four researchers deductively categorised the groups using the seven domains presented in the Rainbow Model for Integrated Care (RMIC), presented in Figure 1 [39,40,41]. Four vertical integration domains (services, professionals, organisations and system) can be distinguished from micro to macro, and relate to different types or levels of care. Two horizontal domains cover functional and normative preconditions. The underlying scope of this model is incorporating that what is best for an individual patient within a population is also best for the population. Therefore, this model covers ‘person-focused and population-based care’ which should lead to better care, better health and lower costs [39,40].

### Researcher characteristics and reflexivity

All the authors have review and/or qualitative research experience and most combine research with clinical care or have experience with IPC. The authors are from different healthcare organisations in the Netherlands and include general practitioners (EB, MP), pulmonologists (BvdB, MdM, MvdH), a program director (AvtH), a senior researcher (LvdB), a physiotherapist (LdZ) and a board chairman (MS) from a tertiary care setting.

## Results

**Figure 2:**
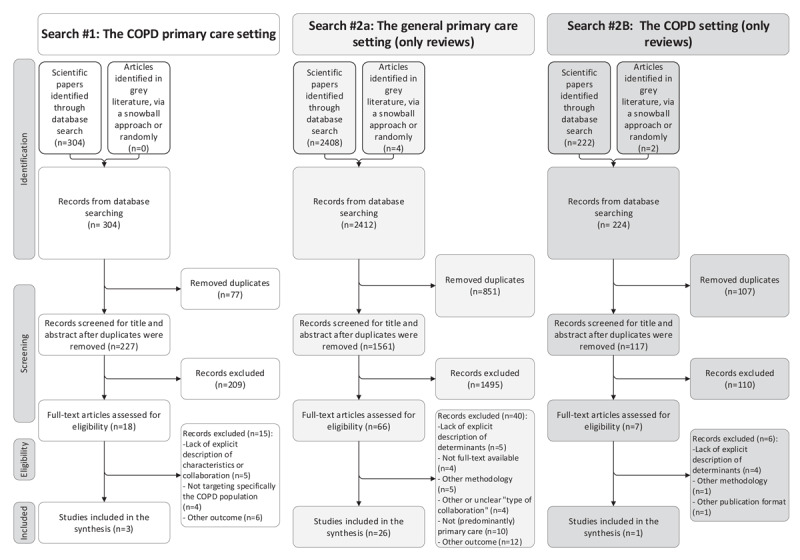
Flow chart illustrating the inclusion of articles from the three searches.

In total, 2940 records were found, unevenly divided over the three searches. After removing duplicates, title/abstract screening, and full text screening, 30 articles remained. Three articles in the COPD primary care setting were selected, twenty-six in the general primary care setting, and one in the COPD setting (Figure 2).

### Study characteristics

Information about the 30 selected articles is listed online in Appendix 4. 20 articles are qualitative research, 10 mixed methods and none purely quantitative. The three articles selected for the primary aim all originate from Canada, with two written by the same research group [43,44]. None of these articles were included in any of the reviews in the subsequent searches. The articles from the general primary care setting originated from 15 different countries, while the article from the COPD setting was from the United Kingdom. Eight of the thirty reviews were systematic reviews [45,46,47,48,49,50,51,52].

### Identified preconditions

Table 1 shows the 44 preconditions of IPC found in the three settings ordered according to the RMIC framework. The COPD primary care setting yielded 32 unique preconditions which cover all domains of the RMIC. Of the preconditions found in the COPD primary care setting, 29 (90%) were similar to those found in the general primary care setting. The general primary care setting yielded 12 additional preconditions.

**Table 1:**
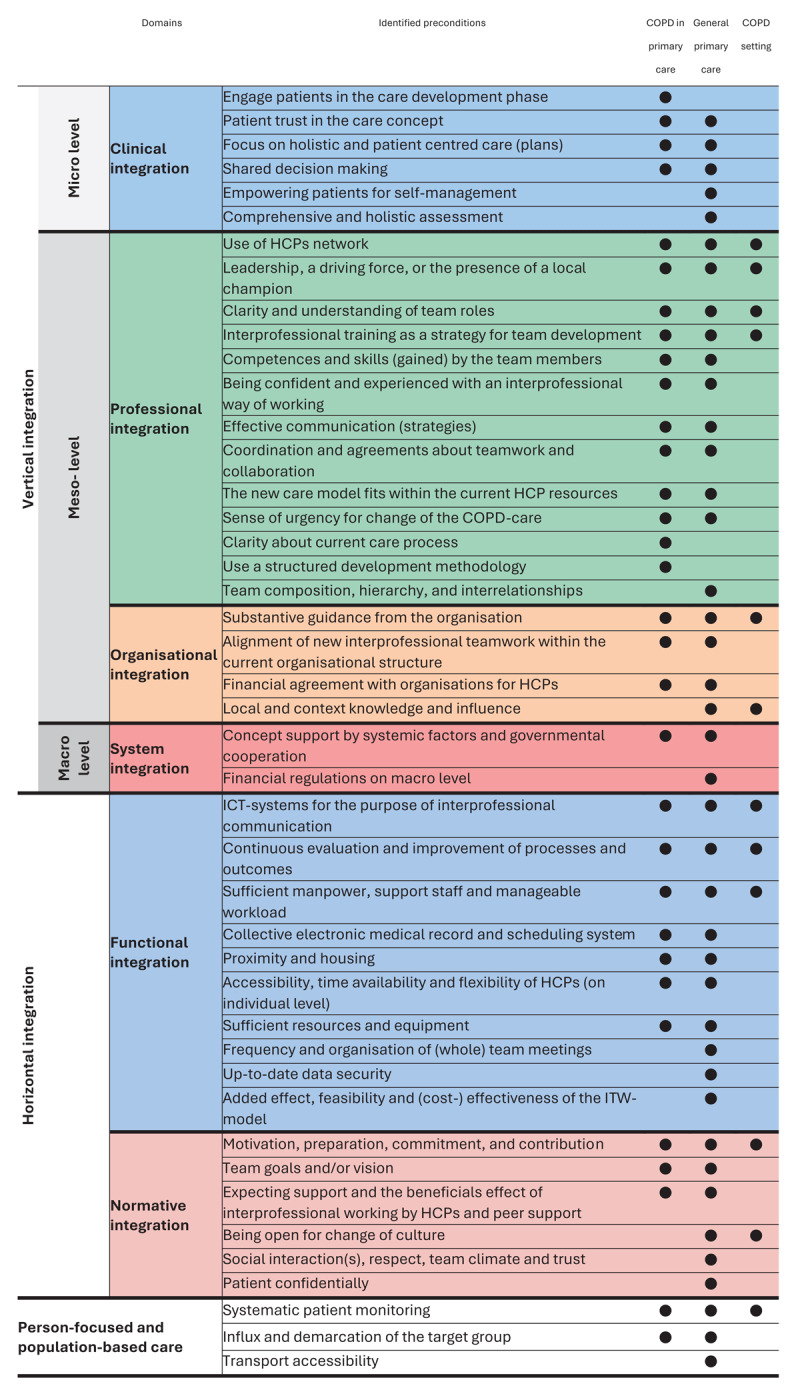
Representation of precondition within its domain per setting. The right-hand 3 columns indicate in which setting(s) the preconditions are found. The colours correspond with the RMIC-domains.

The COPD setting yielded 12 preconditions in total of which 10 (83%) showed overlap with the COPD primary care setting, and all 12 (100%) with the general primary care setting.

### Preconditions per domain

#### Clinical integration

This domain refers to the care trajectory of individual patients and their HCP team [39,40,41]. In most cases this group does not exceed more than five individuals.

The first precondition is including patients when developing an IPC-model as they are the target group or end-users [44]. This can be achieved, for example, through a co-creation approach. Patient integration will lead to greater patient acceptance, which may result in a more appropriate IPC and care [43,45,48]. The more the HCP team target their treatment to the context of the specific patient, the more successful the collaboration is expected to be [43,44,46,47,48,50,53,54,55,56,57,58,59,60,61,62,63]. Many articles emphasize the active role of the patient as a shared decision-making benefit, as well as the benefits of the collaborative teamwork within this domain [43,46,47,53,55,58,64]. According to multiple studies, to make the start of a collaboration easier and assure the best outcomes, individual patients need to be stimulated to take control and actively participate [47,52,53,54,60,61,62,65,66]. Next, a clear patient status praesens identified with a multidimensional assessment facilitates IPC by providing direction for integral treatment goals [54,59,63].

#### Professional integration

Professional integration refers to the group of professionals who execute IPC [39,40,41]. This is a growing number of HCPs over time.

Precondition mentioned for team development was to address the needs of a specific population include using (pre)-existing HCP relationships. Expanding current consultations and/or using pre-existing relationships helps establish the IPC [43,58,59,63,65,67,68]. To adapt to this new way of working IPCs benefit from a driving force, an early adopter who steps up to start up the process and take a leadership role. In the sustaining phase, the presence of a leader remains important, but their focus should shift towards the coordination of care. Many articles note the term ‘case manager’ for this role, and describe this as a key condition for success [43,44,45,46,47,48,49,50,53,54,56,57,58,59,60,61,62,63,64,65,67,68,69,70,71,72].

The case-manager and all HCPs involved must adjust and renew their current role and workflow. Many studies noted a clear division of roles as a precondition for an effective IPC [43,44,45,46,47,48,49,50,53,54,55,56,57,58,59,60,61,62,63,65,66,67,68,69,70,72] which can be achieved by team training. Proposed themes are hosting an interprofessional consultation, defining patient goals, and conflict solving [43,46,47,50,51,52,53,54,55,56,57,58,59,60,61,62,63,64,65,66,68,70,71,72].

Existing individual knowledge and competences such as disease-related information, ability to cope with multifaceted problems, and communication skills are stated as being crucial to the new way of working. If these are absent, they must either be updated or trained [43,45,46,48,52,56,57,58,65,66,69,71]. Pre-existing experience with IPC was considered beneficial [43,45,46,52,55,56,60,61,62,63,64,66] and many papers underline the importance of effective communication with colleagues as being essential for both developing and sustaining IPC [43,45,46,47,48,49,52,53,54,56,57,59,60,62,63,64,66,69,70]. Face-to-face communication is commonly mentioned as the preferred strategy, but in more rural and remote areas, digital alternatives are more convenient and may be a good alternative. Work agreements about teamwork, collaboration, communication, division of tasks, and how duplication of tasks can be avoided, are ideally agreed on and noted in the development phase [43,45,47,50,51,56,58,59,60,61,64,65,69,71]. Preferably, these agreements are based on and aligned with the current resources and way of working. This limits the time investment needed to understand the implementation, as well as the spend on administrative tasks [43,46,52,61]. Another perceived condition is the involved HCPs’ feeling of urgency to change current care as this can boost a new way of working [43,59,72].

Both clarity about the current care process [72], and using a structured development approach [44] are stated as beneficial starting points for initiating changes. The articles do not specifically mention any criteria for the number and type of HCPs in the team, however they do note that a pleasant power dynamics, a low-turnover, a stable and non-hierarchical team are preconditions within the team [46,47,49,50,51,52,53,55,57,58,59,60,61,62,65,66,67,69,70].

#### Organisational integration

Organisational integration describes all the organisations and their involved employees who contribute to the IPC [39,40,41]. Preconditions mentioned for an IPC are engagement, cooperation, and guidance from the involved organisations. Examples given are providing peer-support, discussing ideas, or providing resources [43,44,48,68,70,72].

The new tasks of HCPs involved in the IPC are ideally aligned with the current organisational structure. This requires transparency, minimal differences between the organisations, and providing and encouraging autonomous work [43,45,46,47,48,49,50,53,55,57,58,59,60,61,64,65,66,67,69,70,71]. Furthermore, articles note the value of providing strategic resources, arranging organisational buy-in, and providing access to financial resources as security [43,44,45,46,47,49,50,51,53,55,56,59,60,61,65,66,67,69,70,71,72].

Finally, collaboration should not be a one-size-fits-all approach, and it depends on the local situation and the context in which the care is executed. This can result in differences in organisations involved or in the agreements made between those organisations [56,58,59,67,68].

#### System integration

System integration relates to the fit of an IPC within an existing health care system, so its implementation should work within a regional or national framework [39,40,41]. Health system stability and supportive policy makers are therefore stated as essential preconditions for success [44,52,58,59,60,65,71]. Another frequently mentioned precondition is financial support; either in-kind or by providing funding or reimbursement [45,47,48,50,55,56,59,61,62,63,66,67,69,70,71].

#### Functional integration

Functional integration describes practical preconditions, mechanisms, and tools [39,40,41]. To execute an IPC, the following preconditions have to be met according to multiple studies: ICT systems that facilitate mutual communication [43,58,61,65,68,71,72]; continuing evaluation of processes and outcomes to strive for improvement [43,45,47,48,49,50,53,56,57,58,59,60,61,62,64,65,68,69,71]; and sufficient human resources to manage the workload [43,45,46,47,48,51,52,53,55,56,58,59,60,61,63,65,66,67,68,69,70,71,72].

Another precondition that could help HCPs work effectively together in an IPC is a collective planning system which includes the patient’s medical information [43,45,47,48,49,51,52,53,55,56,58,59,61,64,65,66,69,71]. A shared treatment space or co-location where a case can quickly and easily be discussed can contribute to an effective IPC [45,46,47,48,50,56,58,59,60,61,64,65,66,72]. This also requires flexibility and accessibility of those involved [43,45,46,47,48,49,53,55,58,59,60,61,62,63,64,65,66,67,69,70]. Other preconditions mentioned are sufficient resources and equipment for collaborating, e.g., information leaflets, a system to share test results, computer hardware [48,50,53,55,60,61,62,63,65,66,67,70,72].

IPCs also benefit from whole team meetings to inform each other, discuss the collaboration, and discuss cases. The logistic, organisation and resources of these meetings should match the functional domain [46,47,49,50,51,56,57,58,59,60,61,64,65,69,71]. Moreover, data security has to be guaranteed [58,59,71] as are quality control systems to objectively measure the effect, feasibility and (cost-) effectiveness [45,48,53,61,63,65,71].

#### Normative integration

Normative integration represents the cultural frame and include the values, vision, and a shared mission of HCPs [39,40,41]. Multiple preconditions within this domain are mentioned. Essential to IPC implementation are enthusiastic HCPs who are motivated, prepared, committed and contribute in the process [43,44,45,46,47,49,53,54,55,56,57,59,60,61,62,65,66,67,68,69,70,71,72]. Additional preconditions for this successful new work-form are a shared team goal or vision [44,45,46,47,49,50,51,53,54,57,59,60,61,63,65,67,69,70] and support by the HCPs involved and their direct colleagues [43,47,49,59,61,65,66,67,69,70]. The HCPs need to be open and receptive to change throughout the development, implementation, and sustainability phases [48,59,60,61,65,68]. In most cases, a new way of working needs adjustments and improvements before it reaches a sustainable constant. A conducive team climate with enjoyable social interaction [46,47,49,51,52,53,54,55,58,59,60,61,63,65,67,69,70] and a balanced discussion of cases that protect patient confidentiality [46,47,59] are needed.

#### Person-focused and population-based care

This domain describes how the individual experience of care can be improved, as well as the health of the population and reducing the costs per capita [39,40,41]. Articles note the need for systematic monitoring to adjust the treatment trajectory where necessary, and to improve collaboration by monitoring the population [45,48,63,65,66,68,71,72]. It is important to target the relevant population. On the one hand, the influx should be enough or sufficient to be efficient, on the other hand the population should be clearly demarcated, to provide suitable care. No explicit approach for this is mentioned, only that it should be clear and effective [43,45,46,47,59,60,66]. Finally, transport and logistic options such as parking spaces and accessibility in terms of travel distance should be sufficient to facilitate collaboration [55,56,63].

### Discussion

We identified many preconditions contributing to successful IPCs in the COPD primary care setting, covering all domains of the RMIC framework. Table 1 showed that most of them were found at the professional- and functional integration domain. Preconditions in the organisational and system integration domains were rarely found in the included articles. Whereby we think that in reality there are more. Preconditions identified from secondary literature searches showed large overlap with those found for the COPD primary care setting. The secondary search on IPC in the general primary care setting added 12 preconditions and cover all domains of the RMIC framework. The secondary search on the COPD setting endorsed the found preconditions but did not yield any new preconditions.

The overlap between the search outcomes suggest that the setting is more relevant than the disease to successfully execute IPC. This presupposes that collaboration should be viewed from a (more) generic perspective. The good fit into the RMIC framework presupposes that IPC in the primary care COPD population is complex, needs commitment on all levels, is unpredictable, and should be adjusted to the (local) context and settings, as in other integrated primary care models [41].

The overlap found in our results indicate that an effective treatment approach could be disease transcending. Moreover, health problems identified in patients with COPD are not specific to people with this diagnosis. For example, obesity, inactivity and smoking also occurs in patients with other chronic diseases [7,30,73,74]. To address these TTs, often overlap is found in the involved disciplines. For example, a nurse for advice and smoking cessation, nutritional advice by a dietician, and exercise training at a physiotherapy practice. The structure of collaboration and organisation of care could be similar, however the content of an individual treatment and the addition of certain specialist (pulmonologist for COPD-patients, cardiologist for patients with a cardiovascular disease) could vary.

In practice this can result in embedding a new COPD primary care IPC-approach into an existing collaboration or vice versa. Or otherwise, expending the demarcation of a target group to increase the number of patients that are eligible for this effective care approach. This is emphasized by the number of aged multimorbid people, which is 23% at least and increasing [75,76,77]. This would advocate for a leading role for the general practitioner. A general practitioner is often the overarching HCP in multiple conditions and could be the continuous factor in multiple teams around one patient and could initiate IPC by connecting multiple involved HCPs. Afterwards, case management, communication strategies and goals setting could be discussed within this group.

The low yield of articles specifically related to IPC in COPD primary care was notable, especially because of the frequently mentioned benefits and effectiveness of an IPC in the primary COPD population [18,19,20,78]. Even when broadening the COPD setting for IPC to the secondary and tertiary healthcare settings, we were only able to select one extra citation. This was also unexpected as pulmonary rehabilitation is executed predominantly in secondary and tertiary care settings and has interprofessional care as a key concept [23,24]. Moreover, in 95% of the treatment trajectories, care is provided by at least two HCPs (median 5 HCPs) [21,22,23].

Preconditions were not evenly distributed across the RMIC domains. The system domain is covered by the lowest number of codes. Moreover, these preconditions were described in a generic manner, like ‘good, long term, and adequate funding’ or ‘wider health system stability and supportive policy makers’. This is in clear contrast with preconditions for professional integration, which is covered by the most codes and contains explicit descriptions about the content of interprofessional education, for example: ‘learn about: conflict management, decision making, group process skills’. This uneven distribution between the different RMIC domains is comparable with other studies [79,80,81].

The articles in this review, and therefore the preconditions, show a great degree of heterogeneity, which is helpful to identify preconditions that are highly context-specific e.g., the use of a digital infrastructure may be more important in rural areas. This highlights the importance of understanding the context when developing a programme for an interprofessional team. This is consistent with other literature that describes multiple cases in which the collaboration or the roles and tasks of HCPs differs based on the context in which they operate [82,83]. The heterogeneity is influenced by information exchange modalities, training opportunities, hierarchy in the organisation, the number and type of collaboration partners and healthcare setting [62,67,70,82,83]. Despite the observation of heterogeneity in contexts, multiple generic principles can be applied that fit seamlessly into an integrated care framework.

A next step to promote implementation and add practical value would be a ranking based on relevance of the found preconditions. We could not weigh the preconditions as none of the included studies described the relative importance. To some extent, the presence of a precondition in multiple search strategies could indicate the indisputability of a precondition. We suggest further research could focus on this.

### Strengths and limitations

A main strength of this review is that the primary objective is extended with setting and disease related preconditions, which benefits implementation and places the results in a larger perspective. Second, our findings are the result of a well-described methodological design [32] which includes independent coding, categorising and allocating into code groups and subsequently into domains. These were all performed iteratively with a varied team of researchers from different clinical scientific backgrounds (investigator triangulation), increasing the credibility of our study. Last, the current review is the first to describe the preconditions required for initiating IPCs in primary care for patients with COPD.

We recognize possible limitations, such as missing eligible articles due to the use of example ASReview. To minimalize this chance: 1) two researchers independently screened title/abstracts using different model settings and re-ran the model with new settings to doublecheck for potentially relevant articles in the pool of unscreened articles; 2), we used recommended and accurate model settings [84]; 3) we screened more than 40% of the articles, which is needed to detect at least 95% of the relevant articles [84]; and 4) we anticipated on potential pitfalls and avoided these [37].

Another limitation is the inclusion of only reviews in the second- and third search. If an included review missed or misinterpreted relevant original publications or preconditions, so did we. Even though we were aware of this limitation, including all study designs would yield an unattainable number of articles and would have been redundant to answer the second objective. In all three searches there is potential language bias, which stems from the researchers’ insufficient proficiency in languages other than English and Dutch. This may have resulted in the omission of important preconditions. Especially when these prove to be impactful in specific situations or under local circumstances, missing this precondition is detrimental to implementation.

## Conclusion

In this scoping review, we identified a significant number of preconditions to be considered beneficial when implementing IPC in primary care for patients with COPD. These preconditions were found at all levels of the RMIC-framework.

The identified preconditions for IPC in primary care for patients with COPD turned out to be marginally health care setting and disease dependent as they showed considerable overlap with IPC for other chronic conditions as well as secondary and tertiary care settings. Because of efficiency, it can therefore be recommended to develop and implement IPC in primary care across chronic conditions and with knowledge from already successfully implemented IPC in secondary and tertiary care.

## Additional Files

The additional files for this article can be found as follows:

10.5334/ijic.8991.s1 Appendix 1. PRISMA checlist for scoping reviews.

10.5334/ijic.8991.s2 Appendix 2. Details of the three search strategies.

10.5334/ijic.8991.s3 Appendix 3. ASReview settings and workflow.

10.5334/ijic.8991.s4 Appendix 4. Detailed information about the selected articles.
