# Supplementary material for: Preconditions Contributing to Interprofessional Collaboration in the Management of COPD in Primary Care: A Scoping Review
Source: Int J Integr Care. 2025 Dec 26;25(4):24. doi: 10.5334/ijic.8991 (PMC12742380; doi:10.5334/ijic.8991)
Supplement: Appendix 2. — Details of the three search strategies. [file ijic-25-4-8991-s2.pdf]

| Source | | Terms |
|---|---|---|
| PUBMED | 1 | (interdisciplin*[tiab] OR multidisciplin*[tiab] OR interprofession*[tiab] OR inter-disciplin*[tiab] OR multi-disciplin*[tiab] OR inter-profession*[tiab] OR multiprofession*[tiab] OR multi-profession*[tiab] OR crossdisciplinar*[tiab] or transdisciplinar* OR trans-disciplinar*) |
| | 2 | (patient care team[Mesh] OR patient care team[tiab] OR Team work*[tiab] OR Teamwork*[tiab] OR Care team*[tiab] OR Healthcare team*[tiab] OR Health team*[tiab] OR Collaborat*[tiab] OR network*[tiab] OR management[tiab] OR Interprofessional Relations*[tiab] OR cooperative behavio*[tiab] OR communication[tiab] OR team[tiab] OR cooperati*[tiab]) |
| | 3 | (Determinan*[tiab] OR Keypoint*[tiab] OR facet*[tiab] OR principle*[tiab] OR bulletpoint*[tiab] OR component*[tiab] OR factor* [tiab] OR key discover*[tiab] OR feature* [tiab] or characteristic*[tiab] OR concept* [tiab] OR framework or facilitator [tiab] or barrier*[tiab]) |
| | 4 | (“Primary Health Care”[Mesh] OR Primary care[tiab] OR Primary health service*[tiab] OR Primary health care[tiab] OR Primary healthcare[tiab] OR general practice[tiab] OR family practice[tiab] OR community health[tiab] OR community care) |
| | 5 | (COPD[Mesh] OR Chronic Obstructive Lung Disease[tiab] OR Chronic Obstructive Pulmonary Diseas* [tiab] OR COPD[tiab] OR Chronic Obstructive Airway Disease[tiab] OR Chronic airflow obstructio*[tiab]) |
| | 6 | (2013:2024/03/01[pdat]) |
| | 7 | (english[la] OR dutch[la]) |
| | 8 | (review[pt] OR review*[ti] OR review*[ot]) |
| EMBASE | 1 | ("interdisciplin*" or "inter-disciplin*" or "multidisciplin*" or "multi-disciplin*" or "interprofession*" or "inter-profession*" or "multiprofession*" or "multi-profession*" or "crossdisciplinar*" or "cross-disciplinar*" or "transdisciplinar*" or "trans-disciplinar*").ab,kf,ti |
| | 2 | (“Patient care*” or ”case management” or “collaborative care team” OR “patient care team” OR “Team work*” OR “Teamwork*” OR “Care team*” OR “Healthcare team*” OR “Health team*” OR “Collaborat*” OR “network*” OR “management” OR “Interprofessional Relation*” OR “cooperative behavio*” OR “communication” OR “team” OR “cooperati*”).ab,kf,ti |
| | 3 | (Determinan*:ti,ab,kf OR Keypoint*:ti,ab,kf OR facet*:ti,ab,kf OR principle*:ti,ab,kf OR bulletpoint*:ti,ab,kf OR component*:ti,ab,kf OR factor* :ti,ab,kf OR key discover*:ti,ab,kf OR feature* :ti,ab,kf or characteristic*:ti,ab,kf OR concept*:ti,ab,kf OR framework:ti,ab,kf or facilitator*:ti,ab,kf or barrier*:ti,ab,kf) |
| | 4 | (primary medical care/ or medical care/ or primary health care/ OR Primary care:ti,ab,kf OR Primary health service*:ti,ab,kf OR Primary health care:ti,ab,kf OR Primary healthcare:ti,ab,kf OR general practice:ti,ab,kf OR family practice:ti,ab,kf OR community care OR community health: ti,ab,kf) |
| | 5 | (chronic obstructive lung disease/ or obstructive lung disease/ or Chronic Obstructive Lung Disease:ti,ab,kf OR Chronic Obstructive Pulmonary Diseas* :ti,ab,kf OR COPD:ti,ab,kf OR Chronic Obstructive Airway Disease:ti,ab,kf OR Chronic airflow obstructio*:ti,ab,kf) |
| | 6 | (2013:2024/03/01) |
| | 7 | English or Dutch |
| | 8 | Review |
| Medline | 1 | (interdisciplin* OR multidisciplin*OR interprofession* OR inter-disciplin* OR multi-disciplin* OR inter-profession*OR multiprofession* OR multi-profession*OR crossdisciplinar* OR transdisciplinar OR trans-disciplinar).ab,kf,sh,ti |
| | 2 | (Patient care or case management or collaborative care team OR patient care team OR Team work* OR Teamwork* OR Care team* OR Healthcare team* OR Health team* OR Collaborat* OR network* OR management OR Interprofessional Relations* OR cooperative |

| | | |
|---|---|---|
| | | behavio* OR communication OR team OR cooperati*).ab,kf,sh,ti |
| | 3 | (Determinan* OR Keypoint* OR facet* OR principle* OR bulletpoint* OR component* OR factor* OR key discover* OR feature* or characteristic* OR concept* OR frameworkor facilitator* or barrier*).ab,kf,sh,ti |
| | 4 | (Primary care OR Primary health service* OR Primary health care OR Primary healthcare OR general practice OR family practice OR community health OR community care).ab,kf,sh,ti |
| | 5 | (Chronic Obstructive Lung Disease OR Chronic Obstructive Pulmonary Diseas* OR COPD OR Chronic Obstructive Airway Disease OR Chronic airflow obstructio*).ab,kf,sh,ti |
| | 6 | Last 10 years (search executed on 1st of mart 2024). Extra search only 2013 and 2014 |
| | 7 | (English or Dutch).lg. |
| | 8 | (review or systematic review).pt. |
| Web of science | 1 | TI=interdiscipin* OR AB=interdiscipin* OR TI=inter-discipin* OR AB=inter-discipin* OR TI=multidiscipin* OR AB=multidiscipin* OR TI=multi-discipin* OR AB=multi-discipin* OR TI=interprofession* OR AB=interprofession* OR TI=inter-profession* OR AB=inter-profession* OR TI=multiprofession* OR AB=multiprofession* OR TI=multi-profession* OR AB=multi-profession OR TI=crossdisciplinar* OR AB=crossdisciplinar* OR TI=cross-disciplinar* OR AB=cross-disciplinar* OR TI=transdisciplinar* OR AB=transdisciplinar* OR TI=trans-disciplinar* OR AB=trans-disciplinar* |
| | 2 | ((((((((((((((((((((((((KP=(primary healthcare)) OR KP=(patient care team)) OR TI=(patient care team)) OR AB=(patient care team)) OR TI=(Team work)) OR TI=(care team)) OR TI=(healthcare team*)) OR TI=(health team*)) OR TI=(collaborat*)) OR TI=(network*)) OR TI=(managememt)) OR TI=(Interprofessional Relations*)) OR TI=(cooperative behavio*)) OR TI=(team)) OR TI=(communication)) OR TI=(cooperation)) OR AB=(team work*)) OR AB=(care team)) OR AB=(healthcare team*)) OR AB=(health team*)) OR AB=(collaborat*)) OR AB=(network*)) OR AB=(Management)) OR AB=(cooperative behavio*)) OR AB=(Interprofessional Relations*)) OR AB=(team)) OR AB=(communication)) OR AB=(cooperati*) |
| | 3 | ((((((((((((((((((((((((TI=(Determinan*)) OR AB=(Determinan*)) OR TI=(Keypoint*)) OR AB=( Keypoint*)) OR TI=(facet*)) OR AB=(facet*)) OR TI=(principle*)) OR AB=(principle*)) OR TI=(bulletpoint*)) OR AB=(bulletpoint*)) OR TI=(component*)) OR AB=(component*)) OR TI=(factor*)) OR AB=(factor*)) OR TI=(key discover*)) OR AB=(key discover*)) OR TI=(feature*)) OR AB=(feature*)) OR TI=(characteristic*)) OR AB=(characteristic*)) OR TI=(concept*)) OR AB=(concept*)) OR TI=(framework)) OR AB=(facilitator)) OR TI=(facilitator)) OR AB=(framework)) OR TI=(barrier*)) OR AB=(barrier*) |
| | 4 | ((((((((((((((KP=(primary health care)) OR TI=(primary care)) OR AB=(primary care)) OR TI=(primary health service*)) OR AB=(primary health service*)) OR TI=(primary health care)) OR AB=(primary health care)) OR TI=(primary healthcare)) OR AB=(primary healthcare)) OR TI=(general practice)) OR AB=(general practice)) OR TI=(family practice)) OR AB=(family practice)) OR TI=(community health)) OR TI=(community care)) OR AB=(community care) |
| | 5 | (((((((((AB=(COPD)) OR AB=(Chronic Obstructive Lung Disease)) OR AB=(Chronic Obstructive Pulmonary Diseas* )) OR AB=(COPD)) OR AB=(Chronic Obstructive Airway Disease)) OR AB=(Chronic airflow obstructio*)) OR (TI=(COPD)) OR TI=(Chronic Obstructive Lung Disease)) OR TI=(Chronic Obstructive Pulmonary Diseas* )) OR TI=(COPD)) OR TI=(Chronic Obstructive Airway Disease)) OR TI=(Chronic airflow obstructio*) |
| | 6 | (LA=(English OR Dutch)) |
| | 7 | DOP=(2013-01-01/2024-01-01) |
| | 8 | DT=(Review)) |

| | |
|---|---|
| Search #1 | 1+ 2+ 3+ 4+ 5+ 6+ 7 |
| Search #2a | 1+ 2+ 3+ 4+ 6+ 7+ 8 – (results search 1) |
| Search #2b | 1+ 2+ 3+ 5+ 6+ 7+ 8 – (results search 1) |
