# Supplementary figures and images for: Preconditions Contributing to Interprofessional Collaboration in the Management of COPD in Primary Care: A Scoping Review
Source: Int J Integr Care. 2025 Dec 26;25(4):24. doi: 10.5334/ijic.8991 (PMC12742380; doi:10.5334/ijic.8991)

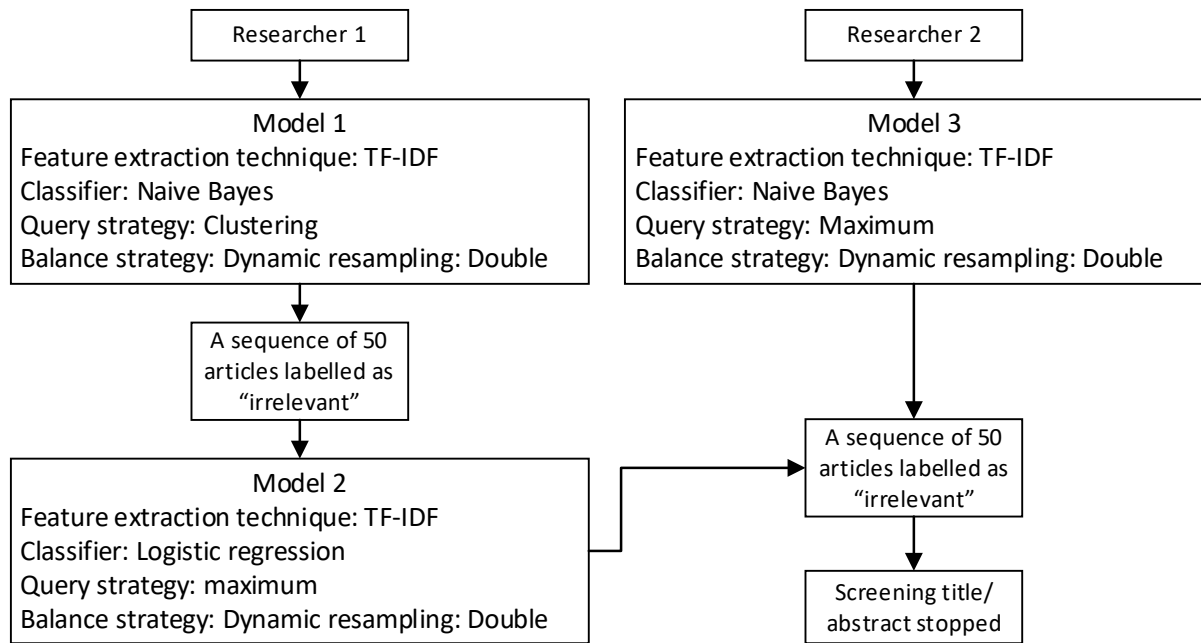

Supplement: Appendix 3. — ASReview settings and workflow. [file ijic-25-4-8991-s3.pdf]
