# Supplementary material for: Preconditions Contributing to Interprofessional Collaboration in the Management of COPD in Primary Care: A Scoping Review
Source: Int J Integr Care. 2025 Dec 26;25(4):24. doi: 10.5334/ijic.8991 (PMC12742380; doi:10.5334/ijic.8991)
Supplement: Appendix 4. — Detailed information about the selected articles. [file ijic-25-4-8991-s4.pdf]

Literature search	1st author and year of publication	Country of the first author	Design	Aim/objective (in case of multiple aims, only the relevant ones are mentioned)
COPD in the primary care setting	Sibbald et al. 2021 (1)	Canada	A mixed-methods cross-case analysis	To explore interprofessional teams at various stages of implementation to understand barriers and facilitators.
COPD in the primary care setting	Paciocco et al. 2021 (2)	Canada	A qualitative case study	To determine the enabling and impeding factors to implementation and spread of an interprofessional team-based primary care model.
COPD in the primary care setting	Vachon et al. 2022 (3)	Canada	A qualitative descriptive retrospective study	1. What are the perceived problems for improving the quality of primary care services offered to people with COPD in Quebec?<br>2. What are the perceived main root causes explaining these quality problems in the primary care management of COPD in Quebec?
The primary care setting in general	Rawlinson et al. 2021 (4)	Switzerland	Review of systematic reviews (qualitative and quantitative)	To identify barriers and facilitators of Interprofessional collaboration in primary care settings.
The primary care setting in general	O' Reilly et al. 2017 (5)	Republic of Ireland	Integrative review (mixed methods)	What does the published literature tell us about barriers and facilitators to the implementation of interdisciplinary teams in primary care?
The primary care setting in general	Montano et al. 2023 (6)	United States of America	Integrative review (mixed methods)	To identify barriers and facilitators to interprofessional collaborative practice for community-dwelling older adults.
The primary care setting in general	Perron et al. 2022 (7)	Canada	Scoping review (qualitative)	To map interprofessional education and interprofessional practice initiatives implemented to promote interprofessional communication in rural and remote primary healthcare facilities and identify barriers and facilitators to their implementation.
The primary care setting in general	Chow et al. 2019 (8)	Canada	Scoping review (qualitative)	To determine how primary healthcare teams collaborate to deliver comprehensive and integrated dementia care to older adults residing in rural and remote areas.
The primary care setting in general	Overbeck et al. 2016 (9)	Denmark	Systematic review (qualitative)	To perform a systematic review of qualitative studies on the enablers and barriers to implementing collaborative care for patients with anxiety and depression.
The primary care setting in general	McNaughton et al. 2021 (10)	New Zealand	Scoping review (mixed methods)	What enablers of and barriers to interprofessional collaborative practice in primary healthcare are identified?
The primary care setting in general	Sangaleti et al. 2017 (11)	Brazil	Systematic review (qualitative)	To synthesize the best available evidence on the experiences of health professionals regarding teamwork and interprofessional collaboration in primary health care settings.
The primary care setting in general	Threapleton et al. 2017 (12)	China	Rapid scoping review (qualitative)	To identify important domains of integrated care systems for older or frail populations and to concisely present evidence on implementation issues.
The primary care setting in general	Supper et al. 2015 (13)	France	Systematic review (qualitative)	To identify factors facilitating or impeding interprofessional collaboration involving other primary care professionals.
The primary care setting in general	Michielsen et al. 2023 (14)	The Netherlands	Scoping review (qualitative)	To research which competencies are necessary for HCP working in collaborative teams where the focus lies within the concept of person-centred integrated care.
The primary care setting in general	Bookey-Bassett et al. 2017 (15)	Canada	Concept analysis (qualitative)	To report a concept analysis of interprofessional collaboration in the context of chronic disease management, for older adults living in communities.
The primary care setting in general	Wood et al. 2017 (16)	United Kingdom	Systematic review (qualitative)	To uncover what barriers and facilitators have been reported by previous research into Collaborative Care for depression in primary care.
The primary	Mulvale et al.	Canada	Systematic	To identify factors that have been shown to have a

care setting in general	2016 (17)		review and conceptual framework (qualitative)	statistically significant association with collaboration in interprofessional primary care teams.
The primary care setting in general	Montano. 2021 (18)	United states of America	Concept analysis (qualitative)	To provide an operational definition of the concept of interprofessional collaborative practice for community-dwelling older adults.
The primary care setting in general	Sørensen et al. 2018 (19)	Norway	Scoping review (mixed methods)	From the perspective of HCPs working in Norwegian primary care, what are the main organisational, processual, relational and contextual facilitators pertaining to multi-professional collaboration involving GPs?
The primary care setting in general	Song et al. 2023 (20)	United States of America	Integrative review (mixed methods)	To amplify the effectiveness of geriatric workforce development efforts in addressing the challenges and barriers facing interprofessional geriatric practice to provide comprehensive, patient-centred home care.
The primary care setting in general	Grant et al. 2017 (21)	Canada	Narrative review (mixed methods)	to assess the benefits of interprofessional collaboration between dental hygienists and other health care providers in rural Canadian communities.
The primary care setting in general	Peer et al. 2022 (22)	United States of America	Integrative review (qualitative)	1) What factors facilitate effective - of mental health care into primary care clinics? 2) What are the barriers to integrating mental health care into primary care clinics?
The primary care setting in general	Wranik et al. 2019 (23)	Canada	Systematic review (mixed methods)	Among interprofessional teams, what is the influence of team characteristics on team process or primary care goal in the context of primary care reforms in Western publicly funded health care systems and focusing on general care, diabetes, asthma, ischemic heart disease, hypertension, and multi-morbidity care?
The primary care setting in general	Muusse et al. 2023 (24)	The Netherlands	Systematic review (qualitative)	To provide an overview of influencing factors of interprofessional collaboration in multifactorial FPIs for community-dwelling older adults living.
The primary care setting in general	Yutong et al. 2023 (25)	China	Scoping review (mixed methods)	What are the hindrances and facilitators of ICT-based practice of integrated care?
The primary care setting in general	Tan et al. 2020 (26)	Singapore	Systematic review (qualitative)	To uncover the perspectives of various stakeholders towards MDT care, discover new understandings and help inform current practice on MDT care for diabetic patients
The primary care setting in general	Morgan et al. 2015 (27)	New Zealand	Integrative review (qualitative)	To examine the nature of interprofessional collaboration (including interprofessional collaborative practice) and the key influences that lead to successful models of interprofessional practice in primary care teams, as reported in studies using direct observation methods.
The primary care setting in general	Levis-Peralta et al. 2020 (28)	Puerto Rico	Scoping review (qualitative)	Identify what organizational factors have been cited in the existing literature as enabling and/or inhibiting team-based care models for diabetes care in primary care settings and categorize these factors.
The primary care setting in general	Parker et al. 2023 (29)	Australia	Scoping review (qualitative)	What are the barriers and facilitators to the participation and engagement of primary care in shared-care arrangements with community mental health services for preventive care of people with serious/severe mental illness?
The COPD population in general	Kozlowska et al. 2018 (30)	United Kingdom	Narrative review (qualitative)	Focus on barriers and enablers of - between primary and specialist care with consideration of the wider context of changes in the healthcare in the UK. The objective of this review is to inform the development and implementation of new - programmes.

1. Sibbald SL, Ziegler BR, Maskell R, Schouten K. Implementation of interprofessional team-based care: A cross-case analysis. J Interprof Care. 2021;35(5):654-61.
2. Paciocco S, Kothari A, Licskai CJ, Ferrone M, Sibbald SL. Evaluating the implementation of a chronic obstructive pulmonary disease management program using the Consolidated Framework for Implementation Research: a case study. BMC Health Serv Res. 2021;21(1):717.
3. Vachon B, Giasson G, Gaboury I, Gaid D, Noel De Tilly V, Houle L, et al. Challenges and Strategies for Improving COPD Primary Care Services in Quebec: Results of the Experience of the COMPAS+ Quality Improvement Collaborative. International journal of chronic obstructive pulmonary disease. 2022;17:259-72.
4. Rawlinson C, Carron T, Cohidon C, Arditi C, Hong QN, Pluye P, et al. An Overview of Reviews on Interprofessional Collaboration in Primary Care: Barriers and Facilitators. Int J Integr Care. 2021;21(2):32.
5. O'Reilly P, Lee SH, O'Sullivan M, Cullen W, Kennedy C, MacFarlane A. Assessing the facilitators and barriers of interdisciplinary team working in primary care using normalisation process theory: An integrative review. PLoS One. 2017;12(5):e0177026.
6. Montano AR, Cornell PY, Gravenstein S. Barriers and facilitators to interprofessional collaborative practice for community-dwelling older adults: An integrative review. J Clin Nurs. 2023;32(9-10):1534-48.
7. Perron D, Parent K, Gaboury I, Bergeron DA. Characteristics, barriers and facilitators of initiatives to develop interprofessional collaboration in rural and remote primary healthcare facilities: a scoping review. Rural Remote Health. 2022;22(4):7566.
8. Chow AF, Morgan D, Bayly M, Kosteniuk J, Elliot V. Collaborative Approaches to Team-Based Primary Health Care for Individuals with Dementia in Rural/Remote Settings. Can J Aging. 2019;38(3):367-83.
9. Overbeck G, Davidsen AS, Kousgaard MB. Enablers and barriers to implementing collaborative care for anxiety and depression: a systematic qualitative review. Implementation science : IS. 2016;11(1):165.
10. McNaughton SM, Flood B, Morgan CJ, Saravanakumar P. Existing models of interprofessional collaborative practice in primary healthcare: a scoping review. J Interprof Care. 2021;35(6):940-52.
11. Sangaleti C, Schveitzer MC, Peduzzi M, Zoboli E, Soares CB. Experiences and shared meaning of teamwork and interprofessional collaboration among health care professionals in primary health care settings: a systematic review. JBI Database System Rev Implement Rep. 2017;15(11):2723-88.
12. Threapleton DE, Chung RY, Wong SYS, Wong E, Chau P, Woo J, et al. Integrated care for older populations and its implementation facilitators and barriers: A rapid scoping review. Int J Qual Health Care. 2017;29(3):327-34.
13. Supper I, Catala O, Lustman M, Chemla C, Bourgueil Y, Letrilliart L. Interprofessional collaboration in primary health care: a review of facilitators and barriers perceived by involved actors. J Public Health (Oxf). 2015;37(4):716-27.
14. Michielsen L, Bischoff E, Schermer T, Laurant M. Primary healthcare competencies needed in the management of person-centred integrated care for chronic illness and multimorbidity: Results of a scoping review. BMC Prim Care. 2023;24(1):98.
15. Bookey-Bassett S, Markle-Reid M, McKey CA, Akhtar-Danesh N. Understanding interprofessional collaboration in the context of chronic disease management for older adults living in communities: a concept analysis. J Adv Nurs. 2017;73(1):71-84.
16. Wood E, Ohlsen S, Ricketts T. What are the barriers and facilitators to implementing Collaborative Care for depression? A systematic review. J Affect Disord. 2017;214:26-43.
17. Mulvale G, Embrett M, Razavi SD. 'Gearing Up' to improve interprofessional collaboration in primary care: a systematic review and conceptual framework. BMC Fam Pract. 2016;17:83.
18. Montano AR. A concept analysis of interprofessional collaborative practice for community-dwelling older adults. Nurs Forum. 2021;56(2):413-20.
19. Sørensen M, Stenberg U, Garnweidner-Holme L. A Scoping Review of Facilitators of Multi-Professional Collaboration in Primary Care. Int J Integr Care. 2018;18(3):13.
20. Song Y, Jung MY, Park S, Hasnain M, Gruss V. Challenges of interprofessional geriatric practice in home care settings: an integrative review. Home Health Care Serv Q. 2023;42(2):98-123.
21. Grant JC, Kanji Z. Exploring Interprofessional Relationships Between Dental Hygienists and Health Professionals in Rural Canadian Communities. J Dent Hyg. 2017;91(4):6-11.
22. Peer Y, Koren A. Facilitators and barriers for implementing the integrated behavioural health care model in the USA: An integrative review. Int J Ment Health Nurs. 2022;31(6):1300-14.

23. Wranik WD, Price S, Haydt SM, Edwards J, Hatfield K, Weir J, Doria N. Implications of interprofessional primary care team characteristics for health services and patient health outcomes: A systematic review with narrative synthesis. Health Policy. 2019;123(6):550-63.
24. Muusse JSC, Zuidema R, van Scherpenseel MC, Velde SJT. Influencing factors of interprofessional collaboration in multifactorial fall prevention interventions: a qualitative systematic review. BMC Prim Care. 2023;24(1):116.
25. Yutong T, Yan Z, Qingyun C, Lixue M, Mengke G, Shanshan W. Information and Communication Technology Based Integrated Care for Older Adults: A Scoping Review. Int J Integr Care. 2023;23(2):2.
26. Tan HQM, Chin YH, Ng CH, Liow Y, Devi MK, Khoo CM, Goh LH. Multidisciplinary team approach to diabetes. An outlook on providers' and patients' perspectives. Primary Care Diabetes. 2020;14(5):545-51.
27. Morgan S, Pullon S, McKinlay E. Observation of interprofessional collaborative practice in primary care teams: An integrative literature review. Int J Nurs Stud. 2015;52(7):1217-30.
28. Levis-Peralta M, González MDR, Stalmeijer R, Dolmans D, de Nooijer J. Organizational Conditions That Impact the Implementation of Effective Team-Based Models for the Treatment of Diabetes for Low Income Patients-A Scoping Review. Front Endocrinol (Lausanne). 2020;11:352.
29. Parker SM, Paine K, Spooner C, Harris M. Barriers and facilitators to the participation and engagement of primary care in shared-care arrangements with community mental health services for preventive care of people with serious mental illness: a scoping review. BMC Health Serv Res. 2023;23(1):977.
30. Kozlowska O, Lumb A, Tan GD, Rea R. Barriers and facilitators to integrating primary and specialist healthcare in the United Kingdom: a narrative literature review. Future Healthc J. 2018;5(1):64-80.
